# A new form of the mole vole *Ellobius tancrei* Blasius, 1884 (Mammalia, Rodentia) with the lowest chromosome number

**DOI:** 10.3897/CompCytogen.v7i2.5350

**Published:** 2013-06-11

**Authors:** Irina Bakloushinskaya, Svetlana A. Romanenko, Natalia A. Serdukova, Alexander S. Graphodatsky, Elena A. Lyapunova

**Affiliations:** 1 Koltzov Institute of Developmental Biology, Russian Academy of Sciences, Moscow, Russia, 26 Vavilov str. Moscow, 119334, Russia; 2 Institute of Molecular and Cellular Biology Siberian Branch, Russian Academy of Sciences, 8/2 Av. Acad. Lavrent’ev, Novosibirsk, 630090, Russia

**Keywords:** speciation, mole voles, *Ellobius*, Robertsonian rearrangements, chromosome painting, *Cricetidae*

## Abstract

The subterranean mole vole, *Ellobius tancrei*, with aspecific variability in autosomes (2n = 31–54) and unusual sex chromosomes (XX in males and females), represents an amazing model for studying the role of chromosome changes in speciation. New materials from the upper reaches of the Surkhob River in the Pamiro-Alay mountains resulted in the discovery of a new form with 2n = 30. The application of Zoo-FISH and G-banding methods allowed the detection of 13 pairs of autosomes as Robertsonian metacentrics originated after fusions of acrocentrics of an assumed ancestral karyotype of *Ellobius tancrei* with 2n = 54. The sex chromosomes (XX, in both sexes) and one pair of acrocentric autosomes are the only acrocentrics in this karyotype, and the set with 2n = 30 possesses the lowest possible chromosome number among populations of *Ellobius tancrei*.

## Introduction

Naturally occurring chromosome variability is essential for understanding the disputed role of chromosome changes in speciation (White 1978, King 1993). A Robertsonian (Rb) translocation is a chromosome rearrangement involving the centric fusion of two acrocentric chromosomes to form a single metacentric chromosome, and it is one of the most frequent events in mammalian karyotype evolution (Slijepcevic 1998, Ruiz-Herrera et al. 2012). Well-studied cases of extensive Robertsonian variation in species such as the house mouse, *Mus musculus domesticus* Schwarz & Schwarz, 1943 (Gropp et al. 1969, Piálek et al. 2005), the common shrew, *Sorex araneus* Linnaeus, 1758 (Ford et al. 1957, Wójcik et al. 2003), or the mole vole *Ellobius tancrei* (Lyapunova et al. 1980, 2010) have their own specific features. All these species are widely distributed; for example, house mice are distributed all over the globe due to human activity. Mice and shrews include a lot of races within their ranges, but *Ellobius tancrei* has 2n = 54 over most of the distribution range and a great variety in chromosome numbers (2n from 53 to 31), which is restricted to a limited area in the Pamiro-Alay, with only single non-homologous translocations (2n = 53) recorded outside, in the Tien Shan mountains (Lyapunova et al. 1985). An amazing feature of mole voles is their subterranean way of life. Living underground preserves mole voles from predators, restricts spreading, and promotes their sociality. The last two factors may contribute to a rapid fixation of chromosome translocations due to more or less permanent monogamous bonds (our unpublished data).

Thorough analysis based on G-banding revealed a complicated structure of chromosome variability in the Surkhob River valley (Pamiro-Alay), where forms with the same chromosome numbers have different sets of Rb metacentrics. It was concluded that the variability was produced by hybridisation, as well as chain fusions (Bakloushinskaya and Lyapunova 2003). New field explorations and applying a fluorescence *in situ* hybridisation (Zoo-FISH) approach have revealed a case of monobrachial homology in this region too (Bakloushinskaya et al. 2010), and raised a question of natural limits for fusions. In numerous field explorations, we were unable to find additional animals at the area where a single specimen with 2n = 31 was collected (Lyapunova et al. 1980). The surrounding areas are inhabited by the form with 2n = 32, and that is why the specimen was considered by us, for a long time, as a case of a single mutation. The main aim of this study was to investigate suitable mole vole areas in the upper reaches of the Surkhob River and determine the structure of karyotypes for discovered animals.

## Material and methods

Five animals (two females and three males) from two colonies were captured by live traps (Golov 1954) on the northern bank of the Surkhob River in Pamiro-Alay (39°15.37'N, 71°20.59'E, 900 m above sea level) in April, 2010.

Chromosomes from bone marrow (Ford and Hamerton 1956) were prepared from all animals; tissues of three specimens were used for tissue culturing. Fibroblast cell lines were prepared as previously described (Sitnikova et al. 2007). Full sets of paints derived from flow-sorted chromosomes of the field vole *Microtus agrestis* Linnaeus, 1761 (Sitnikova et al. 2007) were used. FISH was performed according to previously published protocols (Yang et al. 1999; Graphodatsky et al. 2000). G-banding was carried out for all metaphase chromosomes prior to FISH using trypsin treatments (Seabright 1971).

Images were captured using VideoTesT-FISH 2.0. and VideoTesT-Karyo 3.1. (VideoTesT) or Case Data Manager 6.0 (Applied Spectral Imaging Inc., ASI) software with either a ProgRes CCD (Jenoptik) or ASI CCD camera, respectively, mounted on an Axioskop 2 plus (Zeiss) microscope with filter sets for DAPI, FITC, and rhodamine. Hybridisation signals were assigned to specific chromosome regions defined by GTG-banding patterns previously photographed and captured with the CCD camera. All images were processed using PaintShop Photo Pro X2 (Corel).

## Results

**Figure 1.**
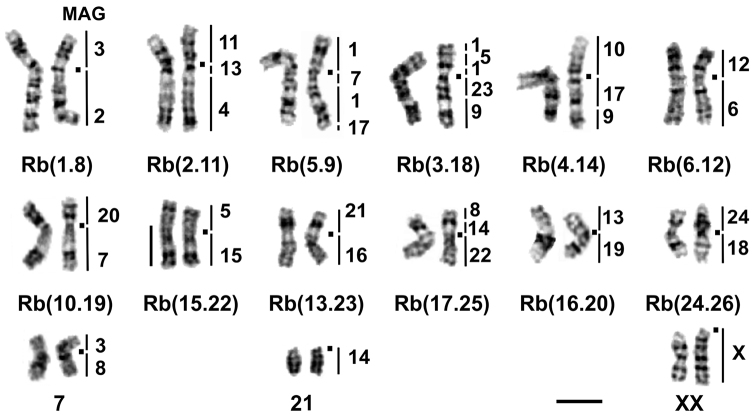
G-banded karyotype of *Ellobius tancrei*, 2n = 30 (25618 ♂). The chromosome nomenclature follows Bakloushinskaya et al. (2012). Black squares mark the positions of centromeres. Vertical black bars and the numbers beside them mark the localisation of *Microtus agrestis* (MAG) chromosome segments. Bar = 10 μm.

**Figure 2.**
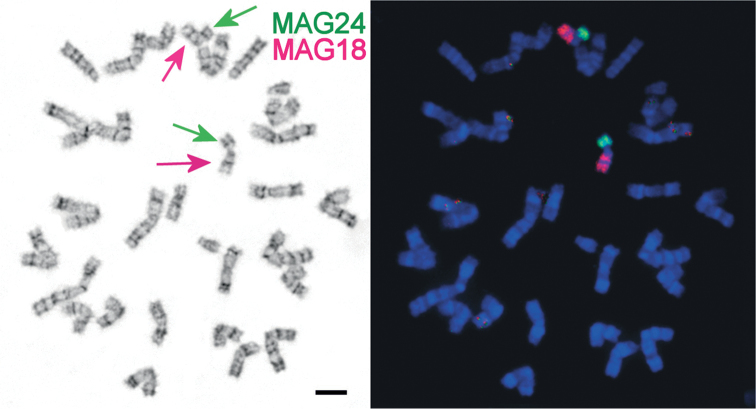
**a** G-banded *Ellobius tancrei*, 2n = 30 (25601 ♀) metaphase spread. **b** the same spread, fluorescent *in situ* hybridisation (Zoo-FISH) of *Microtus agrestis* (MAG) chromosome 18 (red) and 24 (green) on Rb metacentrics (red and green arrows). Bar = 10 μm.

We analysed the structure of karyotypes obtained by direct methods from bone marrow and from cultures. It is known that spontaneous chromosome aberrations may appear in cell cultures (Clare 2012), so it was necessary to control the karyotype structure by a direct method. Karyotypes of all animals contained 30 chromosomes (Fig. 1), and at least 30 plates were counted for each specimen. One pair of submetacentric chromosomes (N 7) is typical for *Ellobius tancrei* and distinguishes it from the chromosomally stable sibling species *Ellobius talpinus* Pallas, 1770. As we reported recently, these chromosomes obtained evolutionary new centromeres (Bakloushinskaya et al. 2012). There are also 12 pairs of Rb metacentrics, one pair of acrocentric chromosomes, and the sex chromosomes XX, which are acrocentric in both sexes. Each of the fifteen chromosome painting probes of the field vole, *Microtus agrestis*, (MAG 2, 4, 6, 10–13, 15, 16, 18–24) delineated one region in the *Ellobius tancrei*, 2n = 30 karyoform; eight probes (MAG 3, 5, 7–9, 13, 14, 17) each delineated two chromosome segments; and the MAG 1 probe delineated four chromosome segments. The only MAG X probe showed signals on both male and female X chromosomes; the MAG Y probes has not produced any specific signal. In total, the 21 MAG autosomal probes revealed 35 conserved segments in the genome, which corresponds to the genome composition of typical *Ellobius tancrei*, 2n = 54 (Bakloushinskaya et al. 2012). The comparison revealed a complete homology between acrocentrics and corresponding Rb metacentrics. The smallest Rb metacentric [Rb(24.26), Fig. 1] has never been detected in karyotypes of other chromosomal forms, including the form with the low chromosome number, 2n = 32, which inhabits the northern bank of the Surkhob River (Bakloushinskaya and Lyapunova 1990). Acrocentric chromosomes involved in the translocation were determined by G-banding as chromosomes number 24 and 26, according to the new nomenclature developed for *Ellobius tancrei*, 2n = 54 (Bakloushinskaya et al. 2012), and confirmed by applying Zoo-FISH probes MAG18 and MAG24 (Fig. 2).

## Discussion

The analysis of the spatio-temporal variation in the structure of a chromosomal polymorphism zone in *Ellobius tancrei* with an interval of 25 years showed that the distribution of chromosomal forms within the area has not changed during this period, except for a small range expansion of a low-chromosomal form (2n = 32) at the western boundary (Lyapunova et al. 2010). Discovery of the chromosome form with 2n = 30 expanded the limits for chromosome rearrangements to the maximal number of fusions in *Ellobius tancrei*, and also moved a border of the chromosomal polymorphism zone to the East, closer to the *Ellobius alaicus* Vorontsov et al., 1969 range. The form with 2n = 30 may be considered as the lowest possible chromosome number for *Ellobius tancrei*, because in such a karyotype only the sex chromosomes (XX, in both sexes) and one pair of acrocentric autosomes remain not rearranged. To date, whole-arm reciprocal translocations (WARTs) have not been recorded in *Ellobius tancrei*. The X chromosomes clearly do not tend to be involved in Rb translocations; a heterozygous 2n = 53 karyotype with an X-autosome Rb-translocation was detected only once in a single female of *Ellobius tancrei* from the Tien-Shan (Lyapunova et al. 1985).

Applying the Zoo-FISH method allows the detection of the homology of translocations, which are only estimated by G-banding. The new karyotype with 2n = 30 contains three Rb translocations (metacentrics 2, 3, 4, Fig. 1) that are homologous to fusions recently described for the 2n = 48 populations inhabiting the northern bank of the Surkhob River, approximately 100 km to the west. Based on these data, we suggest a common origin for these populations. Independent origin may be suggested for a population from the southern bank of the Surkhob River (2n = 50), which shares monobrachial homology with the 2n = 48 form. Furthermore, a partial homology was revealed by Zoo-FISH in spite of their similar G-banding picture (Bakloushinskaya et al. 2010). This case of hidden variability requires a re-investigation of other known chromosomal forms of *Ellobius tancrei*. The Pamiro-Alay is a mountain system with deep valleys and large rivers; mole voles have a mosaic pattern of distribution there. An existence in small demes with limited possibilities for spreading may provoke inbreeding and fast fixing of chromosome rearrangements (Bush et al. 1977). A subterranean way of living may enhance such a process. Speciation by monobrachial centric fusions is one of the well-documented models for house mice (Baker and Bickham 1986, Nunes et al. 2011). However, in the common shrew, the gene flow may be not affected by even extensive monobrachial homology in a hybrid zone between karyotypic races (Basset et al. 2006, Horn et al. 2012). Additional molecular cytogenetic studies are needed to clarify the homology of different chromosomal forms of *Ellobius tancrei* and determine the role of different chromosome rearrangements in species evolution.
